# DrForna: visualization of cotranscriptional folding

**DOI:** 10.1093/bioinformatics/btad555

**Published:** 2023-09-08

**Authors:** Anda Ramona Tănasie, Peter Kerpedjiev, Stefan Hammer, Stefan Badelt

**Affiliations:** Institute of Discrete Mathematics and Geometry, Technische Universität Wien, Vienna, Austria; Department of Theoretical Chemistry, University of Vienna, Vienna, Austria; Department of Theoretical Chemistry, University of Vienna, Vienna, Austria; Department of Theoretical Chemistry, University of Vienna, Vienna, Austria; Department of Theoretical Chemistry, University of Vienna, Vienna, Austria

## Abstract

**Motivation:**

Understanding RNA folding at the level of secondary structures can give important insights concerning the function of a molecule. We are interested to learn how secondary structures change dynamically during transcription, as well as whether particular secondary structures form already during or only after transcription. While different approaches exist to simulate cotranscriptional folding, the current strategies for visualization are lagging behind. New, more suitable approaches are necessary to help with exploring the generated data from cotranscriptional folding simulations.

**Results:**

We present DrForna, an interactive visualization app for viewing the time course of a cotranscriptional RNA folding simulation. Specifically, users can scroll along the time axis and see the population of structures that are present at any particular time point.

**Availability and implementation:**

DrForna is a JavaScript project available on Github at https://github.com/ViennaRNA/drforna and deployed at https://viennarna.github.io/drforna

## 1 Introduction

Cotranscriptional RNA folding refers to the fact that RNA molecules form and change secondary structures dynamically during transcription. Without proper visualization, the information from cotranscriptional folding simulations on what structures are expected to form is only available in large data files, where important structure motifs can easily be overlooked. This is problematic, as computational predictions are also necessary to interpret experimental data. Methods like cotranscriptional SHAPE-seq (Watters *et al.* 2016) measure the reactivity of particular bases which should correlate with the probability of a base being unpaired, but that is not enough information to know which structure has actually been formed. Similarly, optical tweezers can identify at which chain length sudden folding events happen (Fukuda *et al.* 2020), but it is still necessary to augment the procedure with structure prediction to interpret results.

The stochastic helix-level simulator Kinefold (Xayaphoummine *et al.* 2005) uses ‘RNA Movies’ (Evers and Giegerich 1999) to visualize single folding trajectories. This could in principle be used to view the most occupied structure from an ensemble, but then large structural transitions would appear sudden and unexpected. Alternatively, Heine *et al.* (2008) show movies of dynamically changing energy landscapes during transcription. This approach is specifically designed to visualize output of the coarse-grained cotranscriptional folding software BarMap (Hofacker *et al.* 2010), and it does not visualize the respective secondary structures. The most common visualizations are line plots, where each line corresponds to a structure or a set of structures. Those plots are useful for publications, as they can summarize an extensive computational analysis, but they are not suitable for getting a quick and concise visual report of a simulation result.

DrForna, short for ‘DNA-to-RNA Force-directed RNA’ allows the user to convert an input text file containing the details of a folding simulation into a visualization at multiple levels of detail. For example, secondary structures are plotted in rectangles whose area reflects the occupancy at a specific time point. The front end uses a combined linear and logarithmic time scale to get a good overview of simulation results during and after the transcription process.

**Figure 1.**
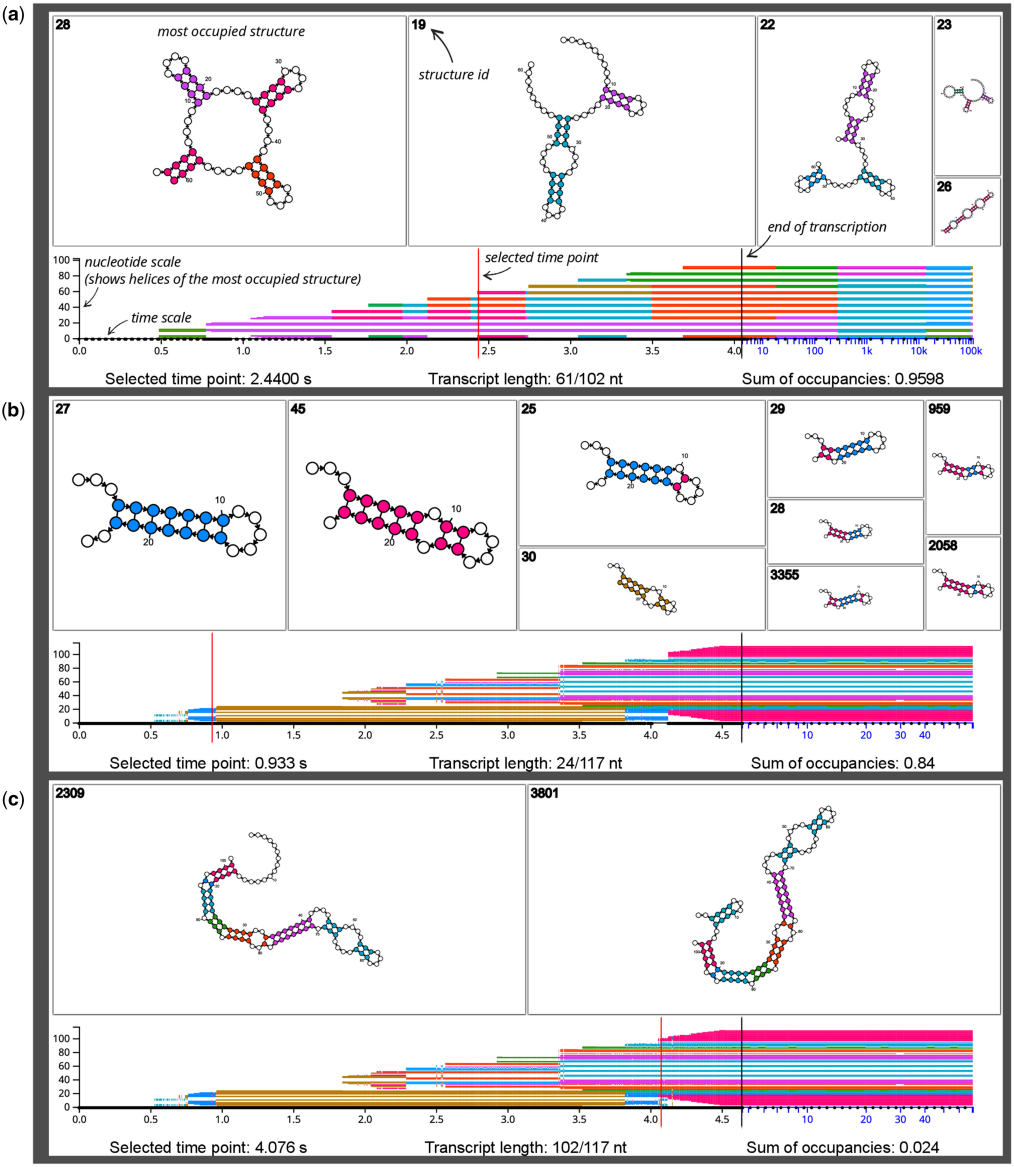
Three SVG downloads from the DrForna cotranscriptional folding visualization application. Additional annotations are provided in italic font. Each example shows a single time point which is written below, and indicated by a red vertical line. The top area shows the individual structures using the default layout of forna (Kerpedjiev *et al.* 2015). The rectangle area is proportional to the occupancy of a structure, and occupancy decreases from left to right. The bottom part of the visualization shows an overview over the other simulation time points. The nucleotide colors of the most occupied structure are plotted along the y-axis to quickly identify regions where the dominant structure changes. The vertical black line is always at 75% of the time scale width and marks the end of transcription, as well as a transition from a linear to a logarithmic scale. This reserves enough space for the often comparatively short time period of cotranscriptional folding compared to a much longer simulation time afterwards. See main text for the interactive features of the JavaScript application. (a) A designed RNA with many cotranscriptionally triggered helix competitions. The image shows a specific time point (2.44 s, at 61 nt length) of a DrTransformer (Badelt *et al.* 2023) simulation, with five competing secondary structures. The sum of occupancies value of 0.9598 indicates that 0.0402 % of the ensemble are not displayed, as the individual occupancies of remaining structures are below a threshold. At the selected time point, the most occupied structure has the form of a clover leaf with four helices. Two of those helices share the same color, as they are perfectly aligned with respect to their imaginary center (31.5 nt). Following the most dominant structures in the bottom panel reveals many cotranscriptional helix competitions. For example, the initially formed green helix is quickly replaced by a pink helix (at 0.75 s), but it appears again long after transcription has ended. Meanwhile, the pink helix remains stable for the whole cotranscriptional folding process. (b) The results of a Kinfold (Flamm *et al.* 2000) simulation of the E.coli SRP RNA. The selected time point shows a competition prior to the formation of the so-called H1 helix, which is believed to play an important part in the cotranscriptional folding process of SRP (Fukuda *et al.* 2020, Yu *et al.* 2021). Our color scheme ensures that small changes in the imaginary center are clearly distinguishable with different colors. Thus, structures with mixed composition of competing helices are easy to identify. The golden struture with ID 30 will become the dominant helix for most of the cotranscriptional folding process. For a more detailed discussion and the biological relevance of these results, see Badelt and Lorenz (2023). (c) The same simulation result as shown in (b), but at a time point where the functional SRP conformation starts to form. Note that the occupancy threshold is chosen to show the two most dominant structures, which combined present only 2.4% of the ensemble.

DrForna provides a platform to identify crucial folding events in a playful manner and to discuss/visualize folding behavior among collaborators. The input file format can be generated from the output of existing cotranscriptional folding software, e.g. in Fig. 1 we show simulations from the heuristic cotranscriptional folding software DrTransformer (Badelt *et al.* 2023) and the stochastic base pair-level simulator Kinfold (Flamm *et al.* 2000). More examples for Kinfold and Kinefold simulations (the latter including pseudoknots) can be seen in Supplementary Section S3, the corresponding RNA sequences can be found in Supplementary Section S4.

## 2 Approach

### 2.1 Input format

DrForna takes a white-space separated value (‘csv-like’) file as input. The header must contain the names ‘id time occupancy structure energy’. The ‘id’ column expects an integer to group structures across different transcript lengths, the ‘time’ column sets a time point for plotting, the ‘occupancy’ column expects a probability of observing the structure in the ensemble at the given time point, the ‘structure’ must be given in dot-bracket notation, and the ‘energy’ is typically given in ${\text{kcal mol}}^{-1}$. A detailed description on specific requirements can be found in Supplementary Section S1, as well as in the online project documentation.

Apart from DrFrona input files described above, the sequence corresponding to a simulation can be uploaded separately. The sequence is then added to the secondary structure plots (not shown in Fig. 1), and it can be easily changed, e.g. to view some constraints used for sequence design, or to annotate specific positions with special characters.

### 2.2 Application frontend

Input text files are uploaded through the web frontend. By default, we provide an example simulation of a sequence that was designed to have many competing secondary structures during transcription. The output combines visualization of ensemble diversity at a specific time point with a high-level overview on changes of most occupied structures over the whole simulation time course, see Fig. 1. The website also provides an additional summary table that shows the currently displayed information from the input file.

#### 2.2.1 Frontend parameters

Besides the upload of simulation files and sequence information, the DrForna frontend provides two additional parameters:

- min-occupancy: This parameter filters structures with low occupancy from the input file to avoid performance problems. For example, at most 100 structures can have occupancy 0.01 at any specific time point. The sum of occupancies is reported for each time point to indicate what fraction of the ensemble is displayed. However, the parameter may remove entire time points from the visualization.
- speed: This parameter adjusts the frame rate of the animation, which can be triggered with the play/pause button. Note that loading structure plots can limit the frame rate and that finding a suitable speed for animation also depends on the number of time points in the input file, as the animation proceeds through each of those time points.

#### 2.2.2 Interactions with the frontend

A user can hover over secondary structures to view their occupancy in numbers, and click on any structure to enlarge it. The cotranscriptional folding process can be watched as an animation using the Play/Pause buttons, but it is also possible to interact through the scale area and hover over a specific area to view folding kinetics in that region of interest. A mouse-click on the scale area freezes/unfreezes the visualization at the currently selected time point, which allows for deeper investigation, image downloads, or screenshots (see Fig. 1).

## 3 Methods

DrForna uses three important algorithms for visualization:

- Secondary structures are produced by the JavaScript package forna (Kerpedjiev *et al.* 2015) and each base pair *i*, *j* is colored depending on the ‘imaginary center’ $c=\frac{i+j}{2}$. We repeat nine colors from the Hue color circle, with the objective that small changes in the imaginary center result in clearly distinguishable colors. Specifically, we chose to traverse the color cycle in steps of $80^{{}^{\circ}}$. As the smallest shift in imaginary centers is 0.5, e.g. a 1-nt bulge, this yields H=⌊c/9⌋+160c mod 360, where the first term ⌊c/9⌋ is added to ensure that color codes cannot repeat for sequences shorter than 360 nucleotides.
- The rectangles for plotting the structures are rendered using the size properties returned by the treemap layout function from the visualization library d3.js (Bostock 2012).
- Time points are skipped dynamically during the mouse-over mode on the interactive scale. Skipping a time point depends on *m*, the maximal number of structures per time point. If time points are selected for shorter than t=5m ms, no output is generated.

See Supplementary Section S2 for a more verbose description of these methods.

## 4 Conclusion

We provide an easy to use, open-source web application for visualizing how structure ensembles change during cotranscriptional RNA folding. In contrast to existing methods, DrForna combines visualization of the dominant structure in the ensemble with a detailed visualization of all currently relevant conformations. The coloring is chosen such that secondary structure motifs can easily be recognized between different plots and the time scale area. As demonstrated in Fig. 1 and Supplementary Section S3, data from various existing simulators can be visualized. However, simulation results from aggregated stochastic trajectories may have to be further processed prior to visualization, otherwise the occupancies of individual structures become too small, and the amount of generated data per time point is too large to be displayed all at once.

## Supplementary Material

btad555_Supplementary_Data

## Data Availability

The data underlying this article are available in the article and in its online supplementary material.
