# Supplementary material for: DrForna: visualization of cotranscriptional folding
Source: Bioinformatics. 2023 Sep 8;39(9):btad555. doi: 10.1093/bioinformatics/btad555 (PMC10504468; doi:10.1093/bioinformatics/btad555)
Supplement: btad555_Supplementary_Data [file btad555_supplementary_data.pdf]

Supplemental material for:
DrForna: visualization of cotranscriptional folding

Anda Ramona Tănasie [1,2], Peter Kerpedjiev [2], Stefan Hammer [2] and Stefan Badelt [2,*]

[1]Institute of Discrete Mathematics and Geometry, Technische Universität Wien (TU Wien).

[2]Department of Theoretical Chemistry, University of Vienna, Austria.

1 DrForna example input

The following text is a slice of valid input for DrFrona. It is a white-space separated value ("csv-like") table, where the header must contain five fields with the names "id time occupancy structure energy".

```
id time occupancy structure energy
6 1.47 1.0000 .((((....((((....))))....))))........  -9.30
6 1.48 1.0000 .((((....((((....))))....))))........  -9.30
9 1.49 0.1494 .........((((....))))....((((....)))). -10.00
6 1.49 0.8506 .((((....((((....))))....)))).........  -9.30
9 1.50 0.2693 .........((((....))))....((((....)))). -10.00
6 1.50 0.7306 .((((....((((....))))....)))).........  -9.30
9 1.51 0.3656 .........((((....))))....((((....)))). -10.00
6 1.51 0.6344 .((((....((((....))))....)))).........  -9.30
```

The value in the energy field has no effect on the visualization, but is displayed in the summary table (see main text Figure 1). The id is used to trace structures over multiple lengths and any id must occur at most once per transcript length. Ideally, structures share the same id if they contain the same base-pairs, that means they differ only in the number of unpaired bases at the 3' end. It is possible to use a different convention for the id field. For example, the BarMap software (Hofacker *et al.*, 2010) uses ids to trace so-called gradient basins in the energy landscape for each transcript length, and the *representative* of a gradient basin may be

*To whom correspondence should be addressed

have a different secondary structure at different transcript lengths. However, if the id does not correspond to a unique set of base-pairs over the whole input file, the colors above the time scale will correspond to the colors of the first structure that appears for each id.

2 Methods for visualization

2.1 Color scheme

As discussed in the main text, we developed a coloring scheme based on the imaginary center of each stem. Suppl. Fig. 1, shows the repetition of nine colors from the Hue color circle using an artificial example that shifts the imaginary center by 0.5. (There exists no sequence compatible with the structures shown in the figure; this is for colorrange demonstrations only.) Although color codes do not repeat within sequences of length shorter than 360 nt, those differences are typically not distinguishable by eye.

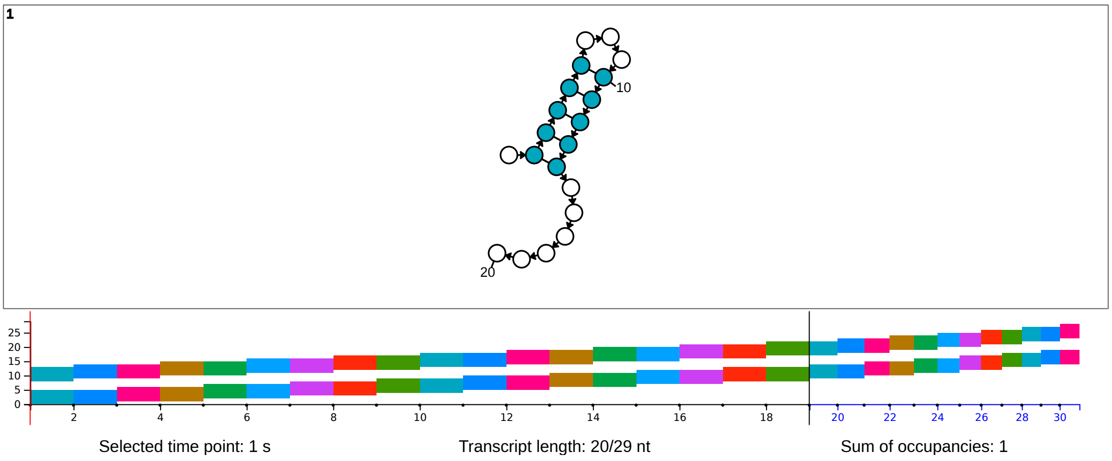

Figure 1: Demonstration of coloring helices by their imaginary centers. Nine colors are repeated, small changes of the imaginary center lead to clearly distinguishable colors. (No sequence is compatible with the example structures plotted here.)

2.2 The treemap layout for structure plots

We use the treemap function from the visualization library d3.js (Bostock, 2012) to adjust the size of rectangles for plotting. As the treemap function expects hierarchical data, the input data is converted into a single level of hierarchy: one parent for each time point to which all data for the time point is connected. Finally, rectangles are placed using the coordinates given

by the treemap function, and contain the associated colored secondary structure as well as the respective ID. The edges of rectangles are shown to ensure that changes in occupancy are always visible, e.g. if a rectangle increases only in width and the secondary structure plot is already scaled to use the maximal height, a change in occupancy would go unnoticed. Note that the size of a rectangle can also increase when the sum of occupancies is smaller than in the previous time step.

2.3 The interactive scale area

Structure plots are generated dynamically when a new time point is selected, which can slow down animations with lots of structural alternatives when many time points are selected in short succession. We use a debounce function to skip timepoints dynamically based on the maximal number of structures m per time point. If time points are selected for shorter than $t = m * 5$ ms, no output is generated. Dynamic scaling based on the total number of structures helps to avoid lag due to computational demands in large input files, where too much data would have to be generated, but also in small files where only few data points are available and thus less time is spent on a single time point when hovering over the scale area.

3 DrForna example visualizations

Suppl. Figures 2 and 3 show DrForna visualization of the stochastic simulators Kinfold (Flamm *et al.*, 2000) and Kinefold (Xayaphoummine *et al.*, 2005). Both plots compare visualization of a single trajectory vs an ensemble generated from 100 trajectories. In practice, it is likely that users may want to generate data from even more individual trajectories, but then some additional post processing (coarse-graining) will be necessary to limit the otherwise overwhelming amount of data. Both figures show the same time point, but secondary structures are quite different. This is because Kinefold is a helix-level simulator which uses a different energy model than the base-pair-level simulator Kinfold. Also, the Kinefold model includes pseudoknotted conformations while Kinfold does not. It is also worth keeping in mind that that the simulation time per nucleotide can have a large impact on the observed secondary structures. In both cases, the structure returned by the single trajectory is not the most occupied structure at 100 trajectories. As the Kinefold model inserts whole helices, the visulization of most occupied structures over time looks smoother than for Kinfold simulations which include base-pair-level stochastic fluctuations.

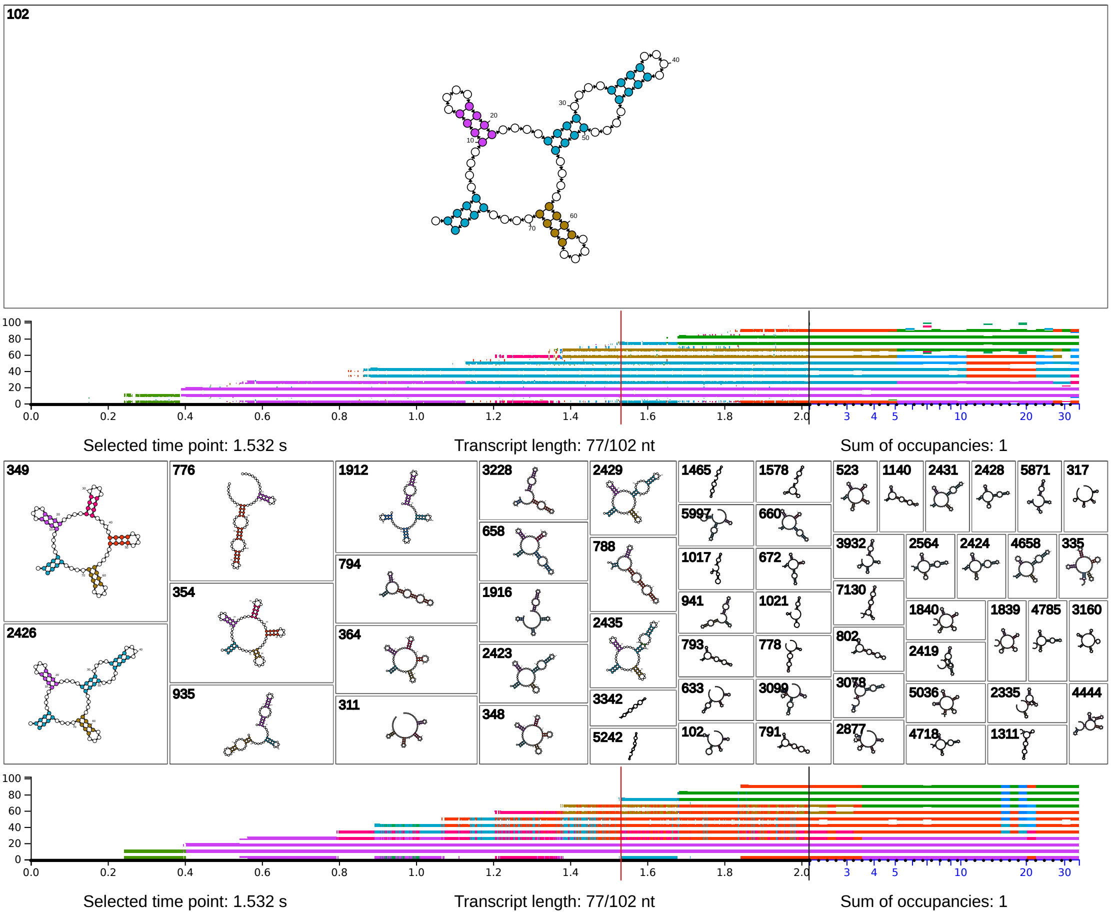

Figure 2: Visualization of a single stochastic Kinfold trajectory, vs an ensemble of 100 trajectories. Simulations use the wrapper script DrKinfold provided in the drconverters repository at: `https://github.com/bad-ants-fleet/drconverters`.

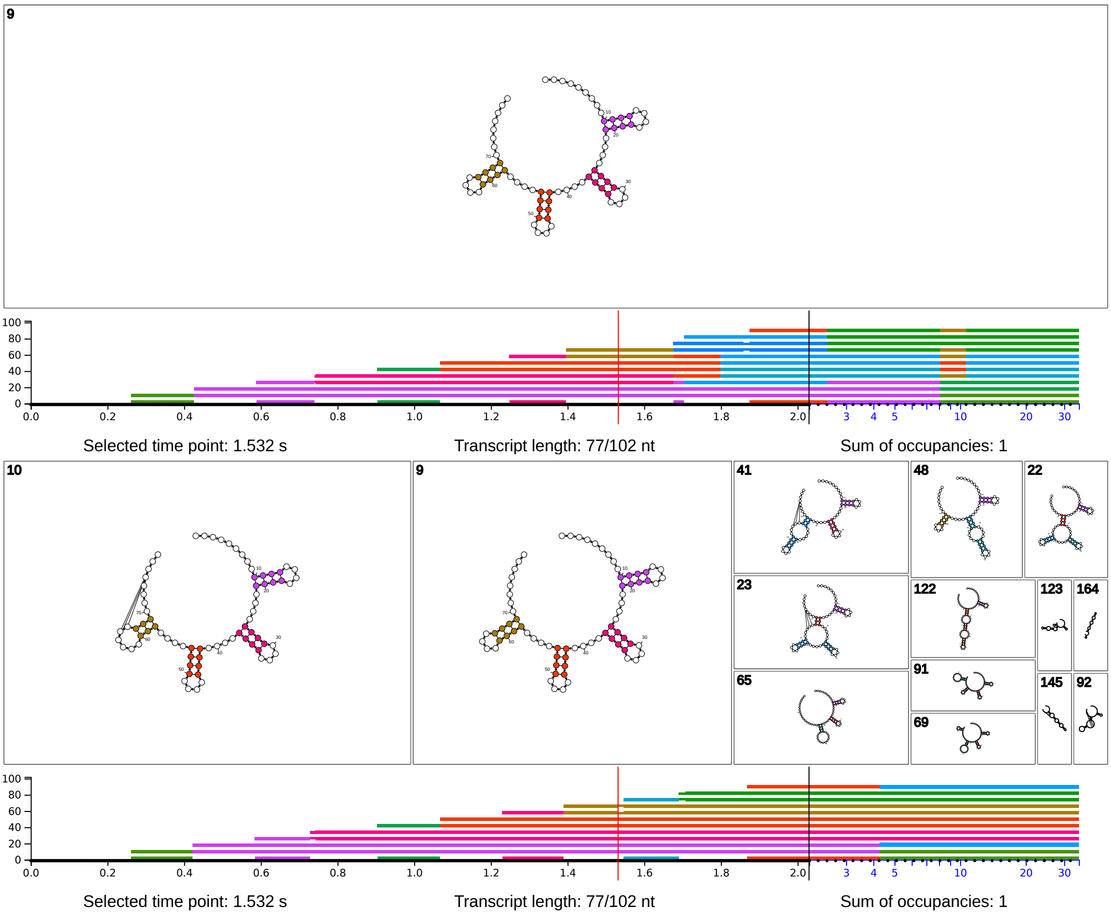

Figure 3: Visualization of a single stochastic Kinefold trajectory, vs an ensemble of 100 trajectories. Simulations use the wrapper script DrKinefold provided in the drconverters repository at: `https://github.com/bad-ants-fleet/drconverters`. Note that pseudoknots are not colored, thus the color codes in the time point selection panel for structure 30 and 9 look identical.

4 RNA sequences

The following sequence was used to generate Figure 1a of the main text, as well as Supp. Figures 2 and 3.

```
>grow
ACUGUCAAUGCGGAAAACCGCUCAAGCGGAAAACCGUAAACGCGGAAACUCGCACACGCG
GAACGUCGCACACGCGGGAAACCGCCAAAGCGGAAACUUGUC
```

The following sequence was used to generate Figure 1b and 1c of the main text.

```
>SRP
AUCGGGGGCUCUGUUGGUUCUCCCGCAACGCUACUCUGUUUACCAGGUCAGGUCCGGAAG
GAAGCAGCCAAGGCAGAUGACGCGUGUGCCGGGAUGUAGCUGGCAGGGCCCCCACCC
```

References

Bostock, M. (2012). D3.js - data-driven documents.

Flamm, C. *et al.* (2000). RNA folding at elementary step resolution. *RNA*, **6**, 325–338.

Hofacker, I. L. *et al.* (2010). BarMap: RNA folding on dynamic energy landscapes. *RNA*, **16**, 1308–1316.

Xayaphoummine, A. *et al.* (2005). Kinefold web server for RNA/DNA folding path and structure prediction including pseudoknots and knots. *Nucleic Acids Research*, **33**(suppl 2), W605–W610.
